# Supplementary material for: Açaí (Euterpe oleracea Mart.) Seed Oil Exerts a Cytotoxic Role over Colorectal Cancer Cells: Insights of Annexin A2 Regulation and Molecular Modeling
Source: Metabolites. 2023 Jun 25;13(7):789. doi: 10.3390/metabo13070789 (PMC10384432; doi:10.3390/metabo13070789)
Supplement: Supplementary file 1 [file metabolites-13-00789-s001.zip › metabolites-2428570-supplementary.docx]

Açaí (*Euterpe oleracea* Mart.) Seed Oil Exerts a Cytotoxic Role over Colorectal Cancer Cells: Insights of Annexin A2 Regulation and Molecular Modeling

Marcos Antonio Custódio Neto da Silva ^1,2^, Josiane Weber Tessmann ^3^, Kátia Regina Assunção Borges ^2^, Laís Araújo Souza Wolff ^2^, Fernanda Diniz Botelho ^4^, Leandro Alegria Vieira ^4^, Jose Andres Morgado-Diaz ^3^, Tanos Celmar Costa Franca ^4,5^, Maria do Carmo Lacerda Barbosa ^2^, Maria do Desterro Soares Brandão Nascimento ^2^, Murilo Ramos Rocha ^3,^* and João Ernesto de Carvalho ^6,^*

^1^ Faculty of Medical Science, Post-graduation in Internal Medicine, State University of Campinas,
13083-970 Campinas, Brazil; [marcos.antonio@ufma.br](mailto:marcos.antonio@ufma.br)

^2^ Nucleum of Basic and Applied Immunology, Pathology Department, Federal University of Maranhão, 65080-805 São Luís, Brazil; [kareborges@gmail.com](mailto:kareborges@gmail.com) (K.R.A.B.); [laiswolff19@gmail.com](mailto:laiswolff19@gmail.com) (L.A.S.W.);
[carminha13032009@hotmail.com](mailto:carminha13032009@hotmail.com) (M.d.C.L.B.); m.desterro.soares@gmail.com (M.d.D.S.B.N.)

^3^ Cell Structure and Dynamics Group, Cellular and Molecular Oncobiology Program, National Cancer
Institute, 20231-050, Rio de Janeiro, Brazil; [jotessmann@gmail.com](mailto:jotessmann@gmail.com)

^4^ Laboratory of Molecular Modeling Applied to Chemical and Biological Defense (LMCBD), Military Institute of Engineering, 22290-270 Rio de Janeiro, Brazil; fernanda.botelho@ime.eb.br (F.D.B.); [leandrovieira@ime.eb.br](mailto:leandrovieira@ime.eb.br) (L.A.V.); [jmorgado@inca.gov.br](mailto:jmorgado@inca.gov.br) (J.A.M.-D.); [tanosfranca@gmail.com](mailto:tanosfranca@gmail.com) (T.C.C.F.)

^5^ Department of Chemistry, Faculty of Science, University of Hradec Kralove, Rokitansheho 62,
500-03 Kralove, Czechia

^6^ Faculty of Pharmaceutical Sciences, Post-graduation in Internal Medicine, State University of Campinas, 13083-970 Campinas, Brazil

***** Correspondence: author: J.E.C. Rua Candido Portinari, 200-Cidade Universitária, Campinas, SP, 13083-871. [carvalho@fcf.unicamp.br](mailto:carvalho@fcf.unicamp.br) (J.E.d.C.) + 55-13-3521-8123 (J.E.d.C.) and M.R.R. Rua André Cavalcanti, 37-Centro, Rio de Janeiro, RJ, 20231-050. [rochamr.2@gmail.com](mailto:rochamr.2@gmail.com); Tel.: + 55-21-3207-6513 (M.R.R.);

Table S1. Identification of chemical compounds extracts from açaí by LC-ESI-MS.

| Compound | Structure | Calculated *m/z* | Observed *m/z* | Error (ppm) | Parts from açaí |
| --- | --- | --- | --- | --- | --- |
| 1 |  | 291.0863 | 291.0848 | -5.15 | seed and total fruit |
| 2 |  | 449.1078 | 449.1056 | -4.89 | pulp and total fruit |
| 3 |  | 579.1497 | 579.1481 | -2.76 | seed and total fruit |
| 4 |  | 595.1657 | 595.1628 | -4.87 | pulp and total fruit |
| 5 |  | 403.1387 | 403.1371 | -3.96 | seed and total fruit |
| 6 |  | 289.0707 | 289.0695 | -4.15 | pulp and total fruit |
| 7 |  | 301.0707 | 301.0695 | -3.98 | seed and total fruit |
| 8 |  | 317.0656 | 317.0643 | -4.10 | seed and total fruit |
| 9 |  | 449.1078 | 449.1060 | -4.00 | pulp and total fruit |
| 10 |  | 433.1129 | 433.1110 | -4.38 | seed, pulp and total fruit |
| 11 |  | 595.1657 | 595.1632 | -4.20 | pulp and total fruit |
